# Preliminary outcomes of Neuroform Atlas stent-assisted coiling for intracranial aneurysms with small parent vessels

**DOI:** 10.1186/s41016-025-00390-x

**Published:** 2025-02-11

**Authors:** Jingrui Xiao, Tianli Li, Dongdong Wan, Qidi Zhou, Xiaolong Zhao, Zhaolong Zhang, Yixing Xie, Liming Shao, Guoping Liu, Chengjian Sun, Rui Xu

**Affiliations:** 1 Department of Interventional Radiology/ Department of Neuro Intervention, The Affiliated Hospital of Qingdao University, Qingdao, Shandong 266000 China; 2 Department of Interventional Radiology, Weihai Municipal Hospital, Cheeloo College of Medicine, Shandong University, Weihai, Shandong 264200 China

**Keywords:** Intracranial aneurysm, Neuroform Atlas stent, Stent-assisted coiling, Treatment outcome

## Abstract

**Background:**

Although stent-assisted coiling has become a standard approach for treating intracranial aneurysms (IAs), there are limited reports on its safety and effectiveness in parent artery less than 2.5 mm in diameter. This study evaluates the feasibility, safety, and short-term outcomes of using Neuroform Atlas stent-assisted coiling for IAs with small parent vessels.

**Methods:**

This study reviewed and analyzed the clinical data of 50 IAs in 50 patients with a parent artery diameter of ≤ 2.5 mm, treated with Neuroform Atlas stent-assisted coiling at a single center between November 2020 and April 2024. Immediate postoperative angiographic outcomes were assessed using the modified Raymond-Roy classification. Follow-up imaging included computed tomographic angiography (CTA), magnetic resonance angiography (MRA), and digital subtraction angiography (DSA). Clinical outcomes were evaluated using the modified Rankin Scale (mRS).

**Results:**

The procedures achieved a 100% success rate. Immediately after treatment, 24 cases were classified as Raymond-Roy grade I, 11 as grade II, and 15 as grade III. Follow-up angiography in 28 cases revealed three instances of aneurysm recurrence, with a secondary procedure performed in one case. One patient reported poor neurological status, and two cases experienced procedure-related adverse events during telephone or clinical follow-up.

Conclusions the Atlas stent demonstrated favorable outcomes in the treatment of aneurysms in small parent arteries (< 2.5 mm), with a low complication rate. The timely postoperative use of tirofiban may further reduce the risk of ischemic complications.

## Background

Intracranial aneurysms (IAs) are pathological dilations at major branching arteries in the brain, which can lead to severe consequences if ruptured. With the advancement of endovascular methods and treatment techniques, endovascular therapy has gradually become one of the primary treatments for IAs [1–3]. The Neuroform Atlas stent (NAS) is a newly developed stent designed for placement in 2.0–4.5 mm vessels, featuring the ability to navigate through small and highly tortuous vessels [4, 5]. There are few reports on the use of the NAS in small-diameter vessels. This study retrospectively analyzed the clinical data of patients with IAs who received NAS in parent arteries ≤ 2.5 mm in diameter at a single center between November 2020 and April 2024, and presents the initial clinical experience with this stent.

## Methods

### Data collection

We retrospectively analyzed the clinical data of 50 IAs with parent artery diameters of ≤ 2.50 mm, treated with stent-assisted coil embolization using NAS at a single center between November 2020 and April 2024. The included IAs were characterized by small parent artery diameters (≤ 2.50 mm), variation in parent artery diameters, large parent artery angles, and the presence of wide-neck aneurysms. IAs with large parent artery diameters (> 2.50 mm) were excluded. Patients who refuse to participate were also excluded. In this study, the cohort consisted of 20 males and 30 females, with a median age of 62.6 years. Of these, 31 patients had hypertension, 9 had diabetes mellitus, 5 had a history of cerebral infarction, 1 had a history of cerebral hemorrhage, and 2 had atrial fibrillation. The group included 5 cases of ruptured IAs, 45 cases of unruptured IAs, and 3 cases of recurrent aneurysms following treatment. The aneurysm locations included 6 cases in the anterior cerebral artery, 13 cases in the anterior communicating artery, 2 cases in the posterior communicating artery (fetal posterior cerebral artery), 22 cases in the middle cerebral artery bifurcation, 1 case in the posterior cerebral artery, 1 case in the posterior inferior cerebellar artery, 1 case in the vertebral artery, and 4 cases at the basilar tip. There were 30 cases of wide-neck aneurysms, 18 cases of narrow-neck aneurysms, and 2 cases of dissecting aneurysms (Table 1). This study complies with the Declaration of Helsinki, and informed consent was obtained from all patients or their families prior to the procedure.

**Table 1:** Baseline characteristics

Characteristics	Value (*N* = 50 aneurysms)
Sex	
Male	20
Female	30
Age, (Median,years)	62.6
Hypertension	31
Diabetes	9
Cerebral infarction	5
Intracerebral hemorrhage	1
Parent artery diameter(mm)	
< 1	3
1 ~ 2	28
2 ~ 2.5	19
Location	
Anterior communicating artery	13
Middle cerebral artery	22
Fetal posterior cerebral artery	2
Anterior cerebral artery	6
Basilar tip	4
Posterior cerebral artery	1
Vertebral artery	1
Posterior inferior cerebellar artery	1
Initial occlusion class	
Raymond I	24
Raymond II	1
Raymond III	15
Stent-related complication	
Intraprocedural thrombus formation	1
Clinical outcome at discharge	
0	41
1	1
2	3
3	5
Follow-up with angiography	
CTA	3
MRA	10
DSA	28
Recurrence	3
mRS score deterioration	1

### Endovascular treatment

After routine preparation, the patient was placed in a supine position with routine tracheal intubation and general anesthesia. The size, location, morphology and parent artery of the aneurysm were assessed using 3D rotation angiography. 34 patients underwent stent-assisted embolization, and the specific treatment process was as follows: Excelsior XT-17 microcatheter (Stryker, USA) or SL-10 microcatheter (Stryker, USA) was placed to the distal end of the parent artery, and SL-10 microcatheter was placed inside the aneurysm, which can slowly release the NAS to fully cover the neck. Then the aneurysms were filled with coil through SL-10 microcatheter. When the direction of the aneurysm and the parent artery was the same, the stent microcatheter could be released and inserted into the aneurysm through the mesh of the stent. One of them underwent Y-stent-assisted coil embolization.

### Perioperative management

In the perioperative management of unruptured IAs, patients received dual antiplatelet therapy, consisting of oral administration of 100 mg aspirin and 75 mg clopidogrel for at least 3 days before treatment. Dual antiplatelet therapy was continued for 3 months after the procedure. After this period, clopidogrel was discontinued, and long-term aspirin therapy was maintained. If clopidogrel resistance was detected, ticagrelor (90 mg twice daily) was used as an alternative. For ruptured IAs, dual antiplatelet therapy was not administered preoperatively. After the stent was released, Tirofiban was slowly injected through the catheter at a dose of 6-8ml. After the operation, Tirofiban was continuously pumped intravenously 4-8ml/h, and the dose of antiplatelet (300mg aspirin +300mg clopidogrel) was stopped 6h after the operation, and then the double antiplatelet therapy was continued orally for 3 months, and only take aspirin after 3 months. Additionally, perioperative medications were administered, including intravenous nimodipine to alleviate vasospasms and mannitol to reduce intracranial pressure. Lumbar puncture was performed to drain bloody cerebrospinal fluid as part of the treatment.

### Angiographic and clinical outcome

We used the modified Raymond-Roy classification to evaluate the degree of embolization immediately after treatment. Post-discharge imaging, either during outpatient visits or through telephone follow-ups, was conducted 1 to 24 months later. The modified Rankin Scale (mRS) was employed to assess clinical status, with deterioration defined as an increase of ≥2 points on the mRS. CTA, MRA, or DSA were used during follow-up to detect aneurysm recurrence. Perioperative ischemic events include new symptomatic cerebral infarction and stent thrombosis. Hemorrhagic events are defined as intracranial hemorrhage confirmed by CT. Aneurysms with a dome-to-neck ratio of less than 2 or a neck size of 4 mm or greater are classified as wide-neck aneurysms. Aneurysm recurrence is defined as an increase in the volume of contrast media filling within the aneurysm observed on follow-up imaging.

## Results

### Treatment outcomes and complications

**Fig. 1:**
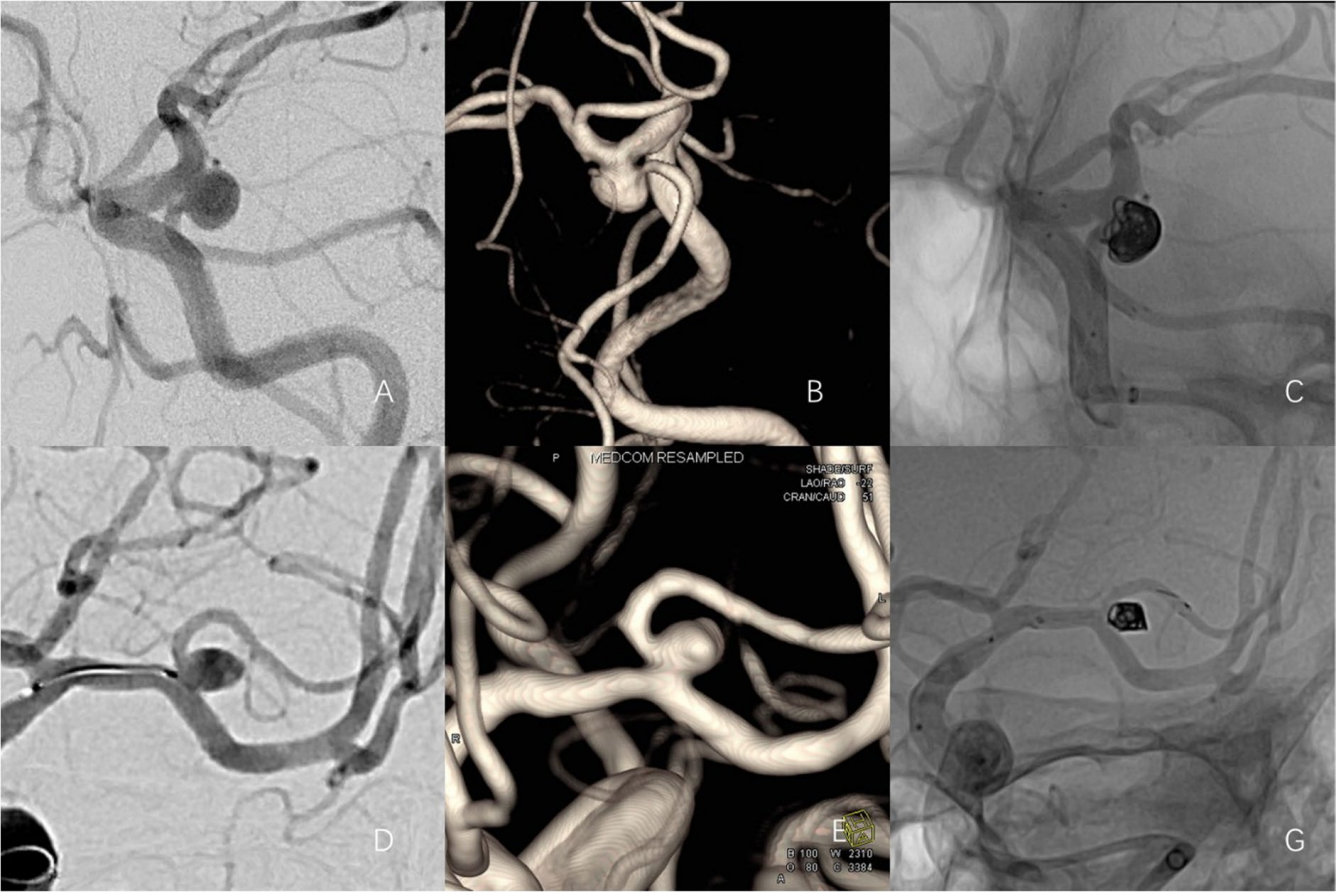
**A**-**C** The left embryonic posterior cerebral artery originates from the aneurysm neck. **D**-**G** Left middle cerebral artery bifurcation aneurysm

Among the 50 aneurysms, 39 were bifurcation aneurysms, and 3 involved a parent artery diameter of less than 1 mm. The stent size was 3.0 mm × 15 mm. There were 28 IAs with a parent artery diameter between 1 and 2 mm (excluding 2 mm). The selected stent sizes were 4.0 mm × 21 mm, 3.0 mm × 15 mm, and 3.0 mm × 21 mm. There were 19 IAs with a parent artery diameter between 2 mm and 2.5 mm. The selected stent sizes were 3.0 mm × 15 mm, 3.0 mm × 21 mm, and 4.0 mm × 21 mm. The selection of stent size should ensure proper vessel attachment without compromising distal blood flow. This means the maximum diameter of the vessel intended for stent placement must be taken into account. For example, when there is a significant difference in diameter between the parent arteries (such as the fetal posterior cerebral artery and the internal carotid artery), the stent size selection should prioritize the diameter of the internal carotid artery (Fig. 1).

The procedure achieved a 100% success rate. Immediately after treatment, 24 cases were classified as Raymond-Roy Grade I, 11 as Grade II, and 15 as Grade III. During the procedure, one case of in-stent thrombosis occurred in a patient with a middle cerebral artery bifurcation aneurysm. After balloon dilation of the occluded segment and intra-catheter administration of tirofiban for thrombolysis, angiography showed restored blood flow. The patient had no new neurological deficits postoperatively, and the mRS score was 2 at discharge. Two patients with basilar apex aneurysms and one with an aneurysm in the A1 segment of the anterior cerebral artery developed transient neurological deficits after treatment. Following intravenous tirofiban infusion, their symptoms improved, and all had an mRS score of 0 at discharge. One patient with a ruptured anterior communicating artery aneurysm developed transient bilateral lower limb weakness after treatment, which was relieved following intravenous nimodipine infusion. The patient was discharged with an mRS score of 3.

### Follow-up outcomes

Postoperative follow-up was conducted on 50 patients. One patient experienced a decline in condition due to a pontine infarction, resulting in an mRS score of 3. Two patients developed new cerebral infarction symptoms, both with an mRS score of 3. Imaging follow-up was completed in 41 patients between 1 and 19 months post-treatment: 3 underwent CTA, 10 had MRA, and 28 had DSA. Aneurysm recurrence was observed in 3 cases; one patient received NAS implantation, forming a Y-shaped stent with coil embolization, while the other two did not undergo further treatment.

## Discussion

Endovascular treatment is currently the primary approach for managing IAs due to its minimally invasive nature and low complication rate [6]. Stent-assisted embolization plays a crucial role in treating wide-neck aneurysms by preventing coil prolapse and covering the aneurysm neck, thereby promoting endothelial growth and reducing the risk of recurrence. The NAS features a hybrid-cell design, combining both open-cell and closed-cell structures. It can be deployed using Excelsior SL-10 (0.0165 in) and XT-17 (0.017 in) microcatheters. Its mesh design offers adequate support while conforming to the vessel, making it highly suitable for distal and tortuous vessels. The open-cell structure at the distal end also ensures excellent performance in parent arteries with significant diameter variations [7].

In a retrospective study, 533 aneurysms in 522 patients were treated with Atlas stent-assisted coil embolization [8]. Neurological complications occurred in 7.3% (38/522) of patients in the early postoperative period, including hemorrhagic complications in 2.3% (12/522) and ischemic complications in 5.0% (26/522). The rate of mRS score worsening was 5.4% (28/522), with a postoperative mortality rate of 0.8% (4/522). During follow-up, the incidence of neurological complications was 1.2% (6/486). The study demonstrated that NAS-assisted coiling for aneurysm treatment is safe, with acceptable morbidity and mortality rates. However, patients with cerebral infarction or Hunt-Hess grades 3–5 may be at higher risk for neurologic morbidity. Independent predictors of neurologic morbidity and mRS score deterioration included procedure duration, stent length, and coil protrusion into the parent artery. Among the participants, 44 patients had anterior circulation aneurysms, and 6 had posterior circulation aneurysms, all with a 100% procedural success rate. Two patients—one with a basilar artery aneurysm and the other with a middle cerebral artery bifurcation aneurysm—developed stent-related cerebral infarctions during follow-up, but no deaths were reported. Of those who underwent angiographic follow-up, 3 cases of aneurysm recurrence were noted. One patient underwent a second procedure involving the placement of a Y-shaped stent and coil embolization, while the other two were monitored. The remaining 25 patients who completed angiographic follow-up achieved complete aneurysm resolution.

### Advantages of NAS in small-diameter vessels

The key to stent implantation in small-diameter vessels lies in the ease of stent positioning, optimal deployment, and good wall apposition. Compared to its predecessor, the Neuroform stent, the ATLAS stent features a smaller delivery microcatheter diameter, making it easier to navigate through narrow and tortuous parent arteries [9]. In the past, treating complex distal aneurysms often required first navigating a microcatheter with a coil to the distal part of the parent artery. This was followed by exchanging a stent-delivering microcatheter using a coaxial system with a microguidewire. This process was intricate and carried risks such as thrombosis and vascular injury. However, the SL-10 and XT-17 microcatheters, with their more flexible tips and ability to be shaped, are easier to maneuver through sharp-angled vessels or aneurysms. Additionally, these microcatheters can be used for subsequent coil embolization, eliminating the need for an extra microcatheter. This approach not only reduces the financial burden on the patient but also decreases the risk of thrombosis associated with multiple catheters [10]. Due to the slim delivery system, the relative motion between the stent delivery catheter and the microcatheter for coil placement is minimized during stent release, ensuring the stability of the microcatheter. The stent’s unique design, with no delivery wire at the stent’s tip, allows for a flat-headed release, meaning the stent delivery catheter does not need to extend far beyond the aneurysm for deployment. The ATLAS stent features a design with an open-cell structure at distal end and a closed-cell structure at proximal end. This open-cell structure distal configuration enables staged expansion, enhancing anchoring even in tapered vessels, while its low shortening rate ensures high precision during release. The stent’s robust radial support and closed-loop proximal structure further increase its stability. Additionally, the open distal and closed proximal design provides adequate support for the coil while enhancing flexibility. The segmental independence of the stent ensures optimal vessel wall apposition, even in tortuous vessels.

### Complications in small-diameter vessels

Treatment complications are a significant limiting factor for stent implantation in small parent arteries, particularly due to perioperative ischemic or hemorrhagic events. Shi et al [11]. reported on the use of LVIS JR stents to treat small parent artery aneurysms, where immediate postoperative angiography revealed aneurysm occlusion rates of Raymond Roy grades I, II, IIIa, and IIIb in 93 cases (71.5%), 24 cases (18.5%), 8 cases (6.2%), and 5 cases (3.8%), respectively. Perioperative complications included 3 cases of acute in-stent thrombosis and 2 cases of severe vasospasm, with no hemorrhagic complications observed. Their analysis suggested that aneurysm size greater than 10.0 mm, a parent artery diameter less than 2.0 mm, and incomplete stent apposition to the aneurysm neck might be risk factors for incomplete aneurysm occlusion. Ozaki and colleagues reported on 49 cases where the ATLAS stent was used for aneurysm-carrying arteries smaller than 2.0 mm [12]. Intraoperative stent thrombosis occurred in 4.1% of patients, and 10.2% experienced symptomatic thromboembolic complications. However, only 4.1% of patients ultimately faced adverse outcomes. The study also demonstrated that middle cerebral artery aneurysms had a higher likelihood of complications compared to aneurysms in other locations. Ozaki and colleagues reported on 49 cases where the ATLAS stent was used for aneurysms in small vessels measuring less than 2 mm in diameter. Intraoperative stent thrombosis occurred in 4.1% of patients, while 10.2% experienced symptomatic thromboembolic complications. However, only 4.1% of patients ultimately suffered adverse outcomes. The study also highlighted that middle cerebral artery aneurysms had a higher risk of complications compared to aneurysms in other locations. In this study, 31 cases involved aneurysms in parent arteries with diameters less than 2 mm, with four patients experiencing transient ischemic complications. Only two patients developed new cerebral infarctions during follow-up: one due to atrial fibrillation, and the other as a result of not adhering to prescribed antiplatelet medication. Importantly, there were no fatalities in this cohort. These favorable outcomes are attributed to the ATLAS stent, which can be deployed via microcatheters with an inner diameter of 0.0165–0.017 inches. Previous stent catheters were not only too rigid to safely reach the target area but also too large in diameter, potentially obstructing blood flow in small vessels, leading to stagnation and subsequent ischemic events. Therefore, stent selection should be based on precise measurements of the parent artery diameter, using both 2D working angle angiography and 3D vascular reconstruction to accurately determine the stent’s distal anchoring point. This study provides preliminary evidence that deploying the ATLAS stent in parent arteries with diameters less than 2 mm is both safe and effective.

Previous reports have also mentioned stent-related ischemic complications during treatment, though adverse outcomes are rare [13–16]. Risk factors for ischemic events include aneurysm rupture with subarachnoid hemorrhage [17] and Y-stent placement [14]. In addition to stent selection, perioperative antiplatelet therapy is crucial. Koh et al. [18] suggested that testing platelet function preoperatively and adjusting antiplatelet medication accordingly may reduce the incidence of thromboembolic events and adverse outcomes without increasing the risk of hemorrhage. Based on our experience, for unruptured aneurysms, administering dual antiplatelet therapy in sufficient doses preoperatively and using tirofiban intraoperatively as needed is effective. In cases of ruptured aneurysms, tirofiban can be used during surgery, followed by dual antiplatelet therapy or a bridging dose postoperatively, depending on the patient’s condition. If a patient develops new neurological deficits, a rapid follow-up CT scan should be performed to rule out intracranial hemorrhage, and intravenous tirofiban should be administered if no hemorrhage is detected.

### Comparison of NAS with others

Currently, there is a lack of large-scale prospective studies on endovascular treatment for IAs in small parent arteries. Most existing studies are retrospective analyses evaluating the safety and efficacy of stent-assisted embolization. This paper provides a review of previous studies (Table 2). The general consensus is that the use of the Atlas stent in small parent arteries is both safe and reliable. In addition to the Atlas stent, LVIS Jr. or LEO stents can also be deployed in small-diameter parent arteries. However, LVIS Jr. and LEO stents are considered braided stents with a high metal coverage rate, which makes their deployment more challenging and increases the risk of thrombosis and perforating artery infarction. Li and colleagues reported a comparative study on the efficacy of LVIS Jr. (69 cases) and ATLAS (66 cases) stents in treating small parent artery aneurysms [19]. Incomplete apposition at the aneurysm neck was observed in one case with the NAS and 11 cases with the LVIS Jr. stent. The perioperative complication rate was 2.22% (3/135). During the six-month follow-up, three cases of aneurysm recurrence were observed in the LVIS Jr. group. Kim and colleagues reported on the use of LVIS Jr. (22 cases) and ATLAS (44 cases) stents in small-diameter parent arteries, achieving a technical success rate of 100% [13]. The perioperative complication rate was 15.2%, with 4.5% of patients experiencing adverse outcomes. During follow-up, 87.9% (51/58) of patients achieved complete aneurysm occlusion. Shen and colleagues reported their experience with the LEO stent, achieving an immediate complete occlusion rate of 82.2% (111/135) postoperatively, and 96.2% (102/106) complete occlusion at six-month follow-up [20]. Treatment-related complications were reported in 10.3% (14/135) of cases, indicating that the LEO Baby stent is reliable for use in small-diameter vessels. Tang et al. evaluated the efficacy of LEO Baby (24 cases) and ATLAS (32 cases) stents in small parent arteries, with ruptured aneurysms accounting for 39.3% (22/56) of cases [21]. Immediate post-procedure occlusion was achieved in 66.1% (37/56) of cases, and the all-cause mortality rate at discharge was 5.4% (3/56). Gross and colleagues compared LVIS Jr. (27 cases) with ATLAS (37 cases) stents, finding that the Raymond 1 occlusion rate was significantly higher in the ATLAS group than in the LVIS group (57% vs. 41%) [16]. Follow-up revealed that the Raymond 1 or 2 occlusion rate was also significantly higher in the ATLAS group (100% vs. 81%), and the rate of in-stent stenosis was significantly lower in the ATLAS group (0% vs. 19%). These results are attributed to the structural features of the ATLAS stent. Across these studies, the ATLAS stent has been shown to be both safe and effective for use in small-diameter parent arteries.

**Table 2:** Related research

Author	Year	Number of cases	parent artery diameter	Stent		Complication	Follow-up with angiography	Recurrence	mRS score deterioration
J. Kim [13]	2021	64	≤ 2	LVIS Jr (22)	Atlas(44)	15.20%	58	2	4.50%
Tomohiko Ozaki [12]	2022	48	< 2	Atlas		4.10%	33	2	4.10%
Osama O.Zaidat [5]	2020	182	-	Atlas		4.40%	153	7	2.20%
Yunan Shen [20]	2022	131	< 2.5	LEO Baby		10.30%	102	2	4.60%
Bradley A Gross [16]	2019	64	-	LVIS Jr (27)	Atlas(37)	3%	25	0	0.00%
Qing-wen Tang [21]	2023	56	< 2	LEO Baby (24)	Atlas(32)	-	40	-	12.50%

### Limitations

There are some limitations to this study. The sample size was relatively small. The retrospective and single-center study design can affect the generalization of results. In angiographic follow-up, the DSA follow-up rate was relatively low. Angiographic follow-up is insufficient to assess long-term treatment outcomes and complication rates. More patients require longer DSA angiographic follow-up. This was not a consecutive case series because patients who met the exclusion criteria were excluded, and thus there may have been selection bias in the patient sampling process. Therefore, prospective multicenter studies are needed.

## Conclusions

In conclusion, the Atlas stent demonstrated favorable outcomes in the treatment of aneurysms in small parent arteries (< 2.5 mm), with a low complication rate. The timely postoperative use of tirofiban may further reduce the risk of ischemic complications. However, the number of cases in this study was limited, and the follow-up angiographic data were insufficient in both quantity and duration. Additionally, a direct comparison with the efficacy and safety of other stents was lacking. Long-term follow-up studies with larger sample sizes are needed to further validate the long-term efficacy and safety of the Atlas stent.

## Data Availability

The datasets used and/or analyzed during the current study are available from the corresponding author on reasonable request.

## Abbreviations

IAs: Intracranial aneurysms
NAS: Neuroform Atlas stent
mRS: Modified Rankin Scale
